# Supplementary material for: WordCommentsAnalyzer: A windows software tool for qualitative research
Source: F1000Res. 2018 Sep 4;7:536. Originally published 2018 May 3. [Version 2] doi: 10.12688/f1000research.14819.2 (PMC6137411; doi:10.12688/f1000research.14819.2)
Supplement: Supplementary file 3 [file f1000research-7-17614-s0002.tgz › b2a79496-4ada-47c4-9682-a99ce867836d.zip!/Eight Iranian Health Journals/Archives of Rehabilitation_16.docx]

© Archives of Rehabilitation. This work is licensed under CC BY-NC 4.0 (https://creativecommons.org/licenses/by-nc/4.0/).

Volume & Issue: دوره 15 - شماره 2

Title: بررسی ارتباط بین ویژگی های فردی و مشخصات کیف مدرسه با گردن درد در دانش آموزان مقطع متوسطه شهر بابل

http://rehabilitationj.uswr.ac.ir/article-1-1518-fa.html

هدف: گردن درد دومین مشکل شایع عضلانی اسکلتی در جوامع بشری است. بر اساس مطالعات، مشخصات فردی و وزن کیف از عوامل زمینه ساز در مشکلات عضلانی اسکلتی دانش آموزان محسوب میشوند. با توجه به وجود زمینه ابتلا به گردن درد، هدف از این مطالعه بررسی تأثیر عوامل خطر زا در دانش آموزان مقطع متوسطه میباشد. روش بررسی: پژوهش حاضر در سال 92-91 بر روی 1000 دانش آموز دبیرستانی بابل انجام شد. دانش آموزان به صورت تصادفی انتخاب گردیدند. مشخصات دانش آموزان و مشخصات کیف و نوع حمل آن و سایر اطلاعات با پرسشنامه جمع آوری شد. از VAS برای بررسی شدت درد استفاده گردید. از ترازو و قدسنج استاندارد برای اندازه گیری وزن و قد استفاده شد. یافته ها توسط نرم افزار SPSS مورد تجزیه و تحلیل قرار گرفت. یافته‌ها: میانگین سنی دانش آموزان 13/1±96/15 سال بود. میانگین وزن کیف و نسبت وزن کیف به وزن بدن به ترتیب 97/0±76/2 کیلوگرم و 87/1±47/4 درصد بود. دانش آموزان دارای گردن درد نسبت به سایرین سن (P<0/001)، قد (P<0/001) و وزن (P=0/002) کمتری داشته و دارای نسبت وزن کیف به وزن بدن بیشتری (P=0/001) بودند. میزان استفاده از کوله پشتی در دانش آموزان دچار گردن درد نسبت به سایرین بیشتر بود (P=0/002). نتیجه‌گیری: نتایج حاضر نشان می دهد ابتلا به گردن درد در دانش آموزانی که ابعاد بدنی کوچکتری دارند بیشتر می باشد. مشخصات کیف نیز می تواند در بروز گردن درد مؤثر باشد. با توجه به اهمیت دانش آموزان به عنوان بزرگسالان آینده کشور بررسی های دقیق در مورد علل شیوع گردن درد در این گروه سنی ضروری بنظر می رسد. همچنین این مطالعه می تواند زمینه ساز مداخلات مناسب درمانی باشد.

Title: سازمان ادراکی و انسجام مرکزی حین پردازش‌های دیداری در کودکان اُتیسم: شواهدی برای از هم گسیختگی ارتباطات کارکردی در مغز اُتیستیک

http://rehabilitationj.uswr.ac.ir/article-1-1406-fa.html

هدف: شواهد گوناگونی، الگوی تغییر یافته کارکردهای ادراکی را در مغز افراد اُتیسم نشان داده­اند، که خود می­تواند مرتبط با و یا علت دیگر علایم تشخیصی این اختلال باشد . در مطالعه حاضر سازماندهی ادراکی دیداری در کودکان اُتیسم موردبررسی قرار گرفت.این پردزاش ­ ها نیاز به انسجام ادراکی اطلاعات و ارتباطات کارکردی بهنجار بین ساختار­های نورونی مغز دارد. روش بررسی: 25 پسر اُتیسم با عملکرد بالا و 25 پسر بهنجار رشدی، از نظر پردازش ­ های ادراک دیداری ارزیابی گردیدند. محرک ­ های دیداری شامل تصاویر گسسته(ناکامل) و کامل واز دو نوع تصاویر اشیاء جاندار و غیرجاندار بود که در صفحه نمایشگر ارائه می­گردید و تکلیف آزمونی شامل نامگذاری سریع و دقیق تصاویر دیده شده بود. جهت مقایسه پاسخ­ها در دو گروه از آزمون t مستقل و برای بررسی تاثیر عوامل درجه تکمیل (وضوح) ونوع تصویر بر پردازش ­ های دیداری نمونه­ها از تحلیل واریانس با اندازه ­ گیری تکراری استفاده شد. یافته‌­ها: اثر تعاملی درجه تکمیل، نوع تصویر و گروه بر پردازش ­ های دیداری نمونه­ها معنادار بود (P<0/05). همچنین کودکان اُتیسم هم در نامیدن تصاویر ناکامل ونیز بازشناسی اشیاء غیرجاندار عملکرد پایین ­ تری داشتند (P=0/000)، در حالیکه هر دو گروه در بازشناسی تصاویر کامل اشیاء جاندار و غیر جاندار یکسان بودند (P=0/597) .از سویی کلیه نمونه­های مطالعه در نامگذاری تصاویر کامل بهتر از گسسته عمل کردند (P=0/000) .   نتیجه‌­ گیری: نقص در بازشناسی تصاویر گسسته اشیاء، بخصوص، غیرجاندار، می ­ تواند نشانگر اختلال در پردازش ­ های مرتبط با انسجام مرکزی اطلاعات دیداری در مغز کودکان اُتیسم باشد. تشخیص این تصاویر در این کودکان به طور معنادار با کودکان هنجار متفاوت بوده که­ می­تواند نشانگر اختلال در سازمان ادراکیِ دیداری و شاهدی برای از هم گسیختگی ارتباطات کارکردی بین نواحی مغز می­باشد.

Title: تأثیر تمرینات ثبات دهنده و تمرینات ایزومتریک حداکثری بر دیدگاه‌های اجتنابی ناشی از درد در بیماران گردن درد مزمن غیر اختصاصی

http://rehabilitationj.uswr.ac.ir/article-1-1348-fa.html

هدف: شواهد روزافزونی وجود دارد که نشان می دهند ترس از درد و ترس از آسیب مکانیسمی پایه ای در پیشرفت درد و ناتوانی ناشی از آن در بیماران است . تمرینات ثبات دهنده گردن روشی است که برای درمان مشکلات و ضایعات ستون فقرات طراحی شده اند و سبب ثبات ستون فقرات گردنی و کاهش آسیب پذیری آن می شود. این مطالعه به منظور تعیین اثربخشی تمرینات اختصاصی ثبات دهنده عضلات گردن نسبت به تمرینات ایزومتریک حداکثری بر دیدگاههای اجتنابی ناشی از درد ، بهبود ناتوانی و درد مزمن گردن انجام شد. روش بررسی: این کارآزمایی بالینی تصادفی کنترل شده روی 50 بیمار مراجعه کننده به مرکز تحقیقات فیزیوتراپی دانشگاه علوم بهزیستی و توانبخشی تهران با تشخیص گردن درد مزمن طی سالهای 92-1391 انجام شد . بیماران به صورت تصادفی در دو گروه 25 تایی تمرین های ثبات دهنده و ایزومتریک حداکثری قرار گرفتند . درد ( رتبه ای ) با مقیاس اندازه گیری دیداری درد ، شدت ناتوانی ( رتبه ای ) با Neck Disability Scale (NDI ) و دیدگاههای اجتنابی ناشی از درد ( رتبه ای ) با Fear Avoidance of Belief Questionnaeir ( FABQ) قبل ، 4 هفته ، 8 هفته و 12 هفته پس از مراجعه ارزیابی شد . برنامه درمانی هر دو گروه شامل 48 جلسه تمرین طی 8 هفته ، هر هفته 6 جلسه و هر جلسه با تکرار تمرین های مشخص بود . از آزمون های آنالیز واریانس ، آزمونهای t مستقل و t زوج برای مقایسه نتایج قبل ، حین و بعد از درمان بین گروهی و درون گروهی استفاده شد. یافته‌ها: میانگین درد در گروه ثبات دهنده از 57/1±16/7 در بدو ورود به 70/0±92/0 در پایان هفته 12 و در گروه ایزومتریک حداکثری از 67/1±12/7 در بدو ورود به 94/0±28/1 در پایان هفته 12کاهش یافت (P=0/482) . میانگین ناتوانی (NDI) در گروه ثبات دهنده از 20/3±60/22 در بدو ورود به 90/0 ±16/11 در پایان هفته 12 و در گروه ایزومتریک حداکثری از 76/2±88/21 در بدو ورود به 48/1±96 /14در پایان هفته 12 کاهش یافت (P<0/001) . میانگین FABQ-W در گروه ثبات دهنده از 84/4±08/35 در بدو ورود به 44/1±08/9 در پایان هفته 12و در گروه ایزومتریک حداکثری از 63/4±76/34 در بدو ورود به 18/2± 44/17 در پایان هفته 12 کاهش یافت (P<0/001) و میانگین FABQ-PA در گروه ثبات دهنده از 26/2±12/20 در بدو ورود به 01/1±26/9 در پایان هفته 12و در گروه ایزومتریک حداکثری از 23/2±08/20 در بدو ورود به 14/1±16/11 در پایان هفته 12 کاهش یافتند (P<0/936). کاهش درد و ناتوانی و کاهش دیدگاههای اجتنابی ناشی از درددر قسمت کار در گروه تمرین های ثبات دهنده نسبت به گروه تمرین های ایزومتریک حداکثری بیشتر بود (P<0/05) . نتیجه‌گیری: این مطالعه نشان داد که هر دو روش تمرین ثبات دهنده و تمرین ایزومتریک حداکثری گردن در بیماران با درد مزمن گردن سبب کاهش درد و ناتوانی و کاهش دیدگاههای اجتنابی ناشی از درد می شوند . منتها تمرینات ثبات دهنده از تاثیر بیشتری برخوردار بودند.

Title: فراتحلیل پژوهش‌های انجام شده در حوزه کاهش آسیب و درمان اعتیاد

http://rehabilitationj.uswr.ac.ir/article-1-1457-fa.html

هدف: هدف از پژوهش حاضر، فراتحلیل پژوهش‌های انجام شده در خصوص کاهش آسیب و درمان است. روش بررسی: در این پژوهش با استفاده از روش فراتحلیلی، مطالعاتی که به کاهش آسیب و درمان پرداخته بودند، گردآوری شدند. برای گردآوری مطالعات، با استفاده از کلیدواژه‌های مرتبط، جستجو در سایت‌ها و پایان‌نامه‌های تحصیلات تکمیلی دانشگاه-ها انجام شد. در نهایت مطالعاتی که با معیارهای ورود به فراتحلیل همخوانی داشتند گزینش شد و اطلاعات مورد نظر برای آزمون فرضیات با پرسش‌نامه معکوسی که توسط نویسنده تهیه شده بود، استخراج شد. از یازده مطالعه گزینش شده، آماره‌های مناسب برای محاسبه اندازه اثر و اطلاعات مورد نیاز دیگر ثبت شد و با نرم افزار CMA2 اندازه اثرها و تجزیه و تحلیل فرضیات محاسبه شد. یافته‌ها: نتایج تحقیق حاضر نشان می‌دهد که در مجموع، متغیرهای مستقل شناسایی شده بر کاهش آسیب و درمان موثر بوده است. مقدار اندازه اثر تلفیق شده ۰٫۸ (۰٫۸d=)، و به لحاظ آماری نیز معنادار است. از آنجایی که این مقدار در دامنه ای بین ۰٫۷ تا ۰٫۸ قرار دارد، طبق دسته‌بندی کوهن، متوسط ارزیابی می شود. نتایج همچنین نشان می دهد متغیرهایی نظیر داشتن مبنای تئوریک، وضعیت ورود (تازه وارد، شش ماه و بیشتر)، میانگین تزریق روزانه، درمان یکپارچه‌نگر، مداخلات درمانی، کیفیت خدمات درمانی، عزت نفس اجتماعی، درمان با متادون و مداخلات درمانی به لحاظ آماری تاثیر معناداری بر کاهش آسیب و درمان داشته است. نتیجه‌گیری: برای دستیابی به کاهش آسیب و درمان، باید وضعیت ورود (تازه وارد، شش ماه و بیشتر)، میانگین تزریق روزانه، درمان یکپارچه نگر، مداخلات درمانی، کیفیت خدمات درمانی، عزت نفس اجتماعی، درمان با متادون و مداخلات درمانی مربوط به اقوام را مد نظر قرار داد.

Title: درد مزمن لگنی در زنان مراجعه کننده به درمانگاه پلویک فلور بیمارستان امام خمینی تهران (1391-1390 )

http://rehabilitationj.uswr.ac.ir/article-1-1289-fa.html

هدف: درد مزمن لگنی عبارت است از هرگونه درد لگن بدون ارتباط با حاملگی، عادت ماهیانه و مقاربت که حداقل شش ماه یا بیشتر به طول انجامیده و علت ساختاری شناخته شده ای برای آن مطرح نشده باشد. این درد می تواند باعث اختلال در فعالیتهای روزانه شده و درنهایت منجر به درمان دارویی و یا حتی جراحی شود. با توجه به کمبود پژوهشهای علمی درمورد این عارضه درکشور این مطالعه با هدف بررسی فراوانی درد مزمن لگنی در زنان مراجعه کننده به درمانگاه پلویک فلور بیمارستان امام خمینی تهران طراحی و اجرا شد. روش بررسی: 516 زن متاهل مراجعه کننده به درمانگاه پلویک فلور در این مطالعه توصیفی از نوع مقطعی شرکت کردند. برای انجام این مطالعه از پرسشنامه ای که علاوه بر اطلاعات جمعیت شناختی شامل سوالاتی در مورد علائم درگیری سیستم های زنان و زایمان، ادراری، گوارشی، عضلانی- اسکلتی و سایکولوژیک بود، استفاده شد. پس از جمع آوری اطلاعات جهت بررسی رابطه متغیرهای کمی و کیفی و درد مزمن لگنی از مدل آماری رگرسیون لجستیک باینری چندگانه استفاده شد. یافته‌ها: 9/27% از زنان گزارش کردند که در حال حاضر درد در ناحیه لگن بدون ارتباط با عادت ماهیانه و مقاربت دارند، درحالی که درصد این درد در طول 12-6 ماه گذشته یا همان درد مزمن لگنی 6/17% بود. همچنین مقاربت دردناک و کمردرد مزمن با درد مزمن لگنی مرتبط بودند (به ترتیب 03/0=P و 0/04=P). بین نوع زایمان و درد مزمن لگنی و سابقه ضربه به ستون فقرات و لگن و درد مزمن لگنی ارتباطی دیده نشد. نتیجه‌گیری: با توجه به شیوع 9/27% درد لگن و 6/17% نوع مزمن آن درجامعه مورد بحث و همینطور همراهی مجموعه ای از علائم سیستم های ژنیکولوژی، ادراری، عضلانی- اسکلتی و گوارشی لزوم پرداختن به آن از طرف سیستم های بهداشت و درمان و نیز انجام مطالعات همه گیر شناسی بر پایه جمعیتی پیشنهاد می شود.

Title: مقایسه تأثیر درمان مبتنی بر آموزش تولید با آموزش حرکات دهانی غیر گفتاری بر گفتارکودکان 6-4 ساله ی مبتلا به اختلال واجی

http://rehabilitationj.uswr.ac.ir/article-1-1206-fa.html

هدف: اختلال صداهای گفتاری از جمله اختلالات شایع گفتار و زبان کودکان محسوب می شود. یکی از درمانهایی که از مدت ها پیش توسط آسیب شناسان گفتار و زبان مورد استفاده قرار گرفته، تمرینات حرکتی دهانی غیر گفتاری می باشد که به عنوان عامل تسهیل کننده قبل یا هم زمان با درمان طیف وسیعی از اختلالات گفتاری به کار می رود، اما داده های تجربی کنترل شده کمی برای بررسی اثربخشی آن وجود دارد. از این رو در این مطالعه  تاثیر مداخله ی درمانی مبتنی بر آموزش تولید با آموزش حرکات دهانی غیر گفتاری در بهبود گفتارکودکان مبتلا به اختلال واجی مقایسه شده است. روش بررسی: این پژوهش مداخله ای شبه تجربی با استفاده از طرح تک آزمودنی با خط پایه مکرر بر روی 6 کودک 6-4 ساله فارسی زبان دارای اختلال واجی با وضعیت دهانی حرکتی طبیعی انجام شد. داده ها با استفاده از آزمون اطلاعات آوایی و پروتکل دهانی حرکتی رابینز-کلی جمع آوری شدند. برای این 6 کودک 3 طرح درمانی با آموزش تولید، تمرینات حرکتی دهانی غیر گفتاری و درمان ترکیبی روی دو صدا با مختصه های واجی متفاوت اجرا شد. نتایج با استفاده از level ,trend & slope analysis با نرم افزار اکسل تحلیل گردید.  یافته‌ها: کودکانی که درمان حرکتی دهانی غیر گفتاری دریافت کرده بودند، تغییری در الگوی گفتاری نشان ندادند اما کودکانی که آموزش تولید دریافت کرده اند 91% و کودکانی که درمان ترکیبی دریافت کرده بودند، 86% بهبودی در صداهای مورد آموزش نشان دادند.  نتیجه‌گیری: نتایج حاصل از این پژوهش از کاربرد تمرینات حرکتی دهانی غیرگفتاری جهت بهبود گفتار کودکان دارای اختلال واجی حمایت نمی کند. همچنین کاربرد این تمرینات پیش از آموزش تولید در قالب درمان ترکیبی با هدف اثر تسهیل کنندگی در بهبود تولید گفتار را رد می کند و آموزش تولید را موثرترین درمان جهت بهبود گفتار کودکان دارای اختلال واجی می داند.

Title: مقایسه اثر اسپلینت داینامیک جدید و استاتیک مرسوم بر میزان درد و توانایی های عملکردی دست در زنان مبتلا به سندروم دکورون

http://rehabilitationj.uswr.ac.ir/article-1-1398-fa.html

هدف: هدف از پژوهش حاضر مقایسه تاثیر اسپلینت داینامیک جدید و استاتیک مرسوم بر میزان درد و توانایی های عملکردی دست در زنان مبتلا به سندروم دکورون بود. روش بررسی: این مطالعه شبه تجربی بر روی 24 بیمار زن مبتلا به سندروم دکورون از نمونه‌های در دسترس در گروه ارتوز و پروتز دانشگاه علوم پزشکی تهران به مدت 2 هفته در سال 92-1391 انجام شد.بیماران به صورت تصادفی به دو گروه 12 نفری تقسیم شدند.به یک گروه از بیماران اسپلینت داینامیک جدید سفارشی ساخت و به گروه دیگر اسپلینت استاتیک مرسوم سفارشی ساخت داده شد. میزان درد وتوانایی‌های عملکردی دست توسط گونه فارسی پرسش‌نامه DASH قبل ونیز دو هفته پس از استفاده از اسپلینت سنجیده شد. همچنین میزان رضایت بیماران از اسپلینت‌ها در انتهای هفته دوم، توسط مقیاس بصری ارزیابی گردید. تحلیل داده‌ها با استفاده از نرم‌افزار آماری spss17 و آزمون‌های آماری تی زوجی و تی مستقل انجام شد. یافته‌ها: نتایج نشان داد که میزان درد و توانایی‌های عملکردی در هر دو گروه پس از استفاده از اسپلینت بهبود‌یافت. مقایسه کاهش درد و افزایش توانایی‌های عملکردی در دو گروه تفاوت معنی‌داری نشان نداد (P>0/05). رضایت بیماران از اسپلینت داینامیک بیشتر بود. (P<0/05) نتیجه‌گیری: افزودن مفصل به مچ اسپلینت اسپایکای شست استاتیک باعث افزایش راحتی و رضایت بیماران می‌گردد و همچنین از نظر بهبود درد و توانایی‌های عملکردی، تفاوت معناداری بین دو گروه مشاهده نمی‌گردد.

Title: بررسی تأثیر روش درمانی پاسخ محور بر توانایی آغازگری کودکان اوتیستیک

http://rehabilitationj.uswr.ac.ir/article-1-1399-fa.html

هدف: اوتیسم یکی از اختلالات فراگیر رشدی است که تعامل اجتماعی، ارتباط کلامی و غیرکلامی و الگوهای رفتاری کلیشه ای را تحت تأثیر قرار می دهد. با توجه به مشکلات آغازگری این کودکان و ضعف مداخلات در ایجاد انگیزه، در این مطالعه سعی کردیم تأثیر روش درمانی پاسخ محور با کاربرد انگیزه را بر توانایی آغازگری کودکان اوتیستیک بررسی نماییم. روش بررسی: در این مداخله کارآزمایی بالینی با گروه کنترل، طبق معیارهای ورود و خروج، 30 فرد اوتیستیک 11-6 ساله که دو به دو از نظر سن و جنس و میانگین طول گفته همتا بودند از کلینیک های مخصوص افراد اوتیستیک شهر همدان انتخاب و بطور تصادفی در دو گروه مداخله و کنترل قرار گرفتند. جلسات بصورت فردی در طی 3 ماه(هفته ای 2 جلسه 60 دقیقه ای) برگزار شد. افراد هر دو گروه قبل و بعد از مداخله با استفاده از پرسشنامه مهارت های ارتباطی کودکان برای ارزیابی توانایی آغازگری ارزیابی شدند. تجزیه و تحلیل داده ها با استفاده از SPSS نسخه 18 توسط آزمون های آمار توصیفی و آزمون کوواریانس انجام یافت. یافته‌ها: یافته های مطالعه نشان داد که طبق آزمون کوواریانس بین دو گروه بعد از مداخله، تغییرات معناداری در توانایی آغازگری (0/001>P) در گروه مداخله وجود دارد. نتیجه گیری: یافته های این پژوهش نشان دهنده‌ی تأثیر روش درمانی پاسخ محور برتوانایی آغازگری کودکان اوتیستیک می باشد و بهتر است در مداخلات درمانی این روش درمانی نیز بکار برده شود.

Title: اعتبار و تکرارپذیری Dual Digital Inclinometer در اندازه گیری زاویه کایفوز پشتی در زنان بالای 45 سال

http://rehabilitationj.uswr.ac.ir/article-1-1334-fa.html

هدف: اندازه گیری دقیق کایفوز پشتی ستون فقرات هم در روند ارزیابی بیماران و هم در سنجش اثر درمانهای مختلف از ارزش بالایی برخوردار است.Dual Digital Inclinometer(DDI) به عنوان ابزاری که می تواند به صورت غیر تهاجمی مقدار زاویه کایفوز را اندازه گیری کند معرفی شده است. امابنا بر اطلاعات موجود تاکنون تنها یک مطالعه به بررسی اعتبار و تکرارپذیری این ابزار درکشورمان پرداخته است. لذا هدف از این مطالعه بررسی اعتبار وتکرارپذیری درون روز وبین روز ابزار DDI دراندازه گیری زاویه کایفوز پشتی ستون فقرات در جامعه زنان بالای 45 سال می باشد. روش بررسی: در این مطالعه متدلوژیک، کایفوز پشتی ستون فقرات توسط ابزار DDI در سه نوبت بر روی 20 زن در دامنه سنی 70-45 اندازه گیری شد.دو اندازه گیری در یک روز به فاصله یک ساعت برای بررسی تکرارپذیری درون –روز و نوبت آخر با فاصله یک روز برای ارزیابی تکرارپذیری بین- روزها صورت گرفت.همچنین مقدارزاویه کایفوز با محاسبه زاویه Cobb بر روی تصاویررادیولوژیکی توسط آزمونگر اندازه گیری شد و اعتبارزاویه به دست آمده با ابزار DDI در مقایسه با آن مورد بررسی قرار گرفت.از آزمون ضریب همبستگی ICC با فاصله اطمینان 95% جهت تجزیه و تحلیل داده ها استفاده شد. یافته‌ها: دربررسی اعتباراندازه گیری کایفوز به دست آمده با ابزار DDI در مقایسه با زاویه Cobb مقدار ICC=0/86 به دست آمد که سطح اعتباربالایی را نشان می دهد. همچنین تکرارپذیری درون روز و بین روز زاویه کایفوز پشتی اندازه گیری شده با ابزار DDI به ترتیب با ضریب همبستگی ICC=0/95 و ICC=0/92 به دست آمد. نتیجه‌گیری: بنابر یافته های این پژوهش، ابزار DDI می تواندبه عنوان وسیله ای معتبر و تکرارپذیر برای اندازه گیری غیرتهاجمی کایفوز پشتی ستون فقرات به کارگرفته شود.
